# Spatial Frailty Survival Model for Multidrug-Resistant Tuberculosis Mortality in Amhara Region, Ethiopia

**DOI:** 10.1155/2019/8742363

**Published:** 2019-01-01

**Authors:** Ashenafi Abate Woya, Abay Kassa Tekile, Garoma Wakjira Basha

**Affiliations:** Department of Statistics, College of Science, Bahir Dar University, P.O. Box 79, Bahir Dar, Ethiopia

## Abstract

Tuberculosis (TB), a disease caused by* Mycobacterium tuberculosis* (MTB), is the main cause of death. It disproportionally affects those living in the different regions of countries and within the region. The aim of this study was to examine spatial variation of mortality and the risk factor of death on multidrug-resistant tuberculosis patients treated in different MDR-TB hospitals of Amhara region. The data for this study was used from multidrug-resistant tuberculosis patients' record charts and analyzed using STATA software. The result of this study shows that 61 (29.47%) of the patients died, and the rest, 146 (70.53%), of the patients were censored at the time of the study. Out of 207 MDR-TB, 146 (70.53%) were males and 61 (29.5%) were females. This study revealed that there was no heterogeneity for death in patients treated in different hospitals. Older patients, therapeutic delay, alcohol use, any clinical complication previously not treated, HIV coinfection, and presence of any chronic disease were the risk factors that influenced the death of multidrug-resistant tuberculosis patients.

## 1. Introduction

Tuberculosis (TB), a disease that killed approximately 2 billion people over the last 200 years, remains a threat to humankind [1]. It disproportionally affects those living in low- and middle-income countries and within countries [1–3]. The most recent global TB report estimated that there were 10.4 million new cases globally and 1.4 million deaths in 2015 [1]. The continent of Africa reports particularly high incidence rates; it accounts for 26% of all TB cases in the world and the highest reported incidence rate of 275 cases per 100,000 population [1]. According to WHO report, 87% of TB burden was found in Ethiopia [4]. Ethiopia is one of these 30 high-burden countries and has been classified as having high burdens of TB, multidrug-resistant TB (MDR-TB), and TB-HIV coinfection [4]. World Health Organization (WHO) reported that Ethiopia is one of the countries known to have a huge burden of TB [1].

Based on the national population survey of Ethiopia conducted in 2010, the prevalence of smear-positive and all forms of TB had estimated 108/100,000 population and 240/100,000 population, respectively [5]. The Government of Ethiopia has incorporated TB control as one of the priority health program packages in the country [5]. However, TB and MDR-TB still constitute a major public health problem in Ethiopia with high variation from region to region and within the region. Thus, the aim of this study was to assess the survival time and predictors of mortality among patients under treatment of multidrug-resistant tuberculosis. In this study we identify the correlation between multidrug-resistant tuberculosis patients death among districts of hospitals. The spatial frailty model was used, which can take the presence of the death difference among patients of the district.

## 2. Methodology

### 2.1. Study Design

This study was a retrospective study design. Participants of the study were recruited at the admission point of the MDR-TB and followed up during their stay in the unit, with note-taking of all significant clinical events. The data considered in the study belongs to a patient of tuberculosis who started multidrug treatment at different hospitals before six months of February, 1.2018.

### 2.2. Study Area

The study was conducted on multidrug-resistant tuberculosis in different hospitals of Amhara region which is located in the north west part of Ethiopia.

### 2.3. Data Source

The data were obtained from different hospitals of Amhara region that have multidrug resistance tuberculosis patients (Debre Tabor Hospital, Gondar Teaching Hospital, and Debre Markos Hospital). The hospital location or district patients were the random effect for this study

### 2.4. Data Collection

Data was collected by the nurses from patients' record charts using a pretested standard questionnaire and follow-up data collection form. The data were collected from February to April in 2018.

### 2.5. Data Management and Analysis

The collected data was coded to maintain confidentiality, then entered into Epi Info, and analyzed using STATA software version 14.

### 2.6. The Study Variable

The response variable in the study was survival time of MDR-TB patients. The survival time of MDR patients was a treatment continued until the date of death or censor occurred. The date of data was obtained from the patient's history charts. Sociodemographic factors, clinical factors, and districts of hospitals were the independent variable in this study.

### 2.7. Statistical Model

In this study, we considered survival models for the multidrug resistance TB dataset which are spatially arranged. Such a spatial arrangement of the strata can be used in geostatistical modeling of the strata. Then, spatial frailty model was applied to analyze multidrug resistance TB. In this study, gamma shared frailty was used with different baseline distributions. Using STATA software, the hazard rate of death and the significance of factors were identified.

Cox proportional hazard model is presented in the form

(1) $$\begin{array}{c} h\left(\;t_{ij},x_{ij}\;\right)=h_{o}\left(\;t_{ij}\;\right)\exp\left(\;\beta^{T}x_{i}\;\right) \end{array}$$

where *t*_*ij*_ is the time to death or censoring for individual *i* in the district *j*; *i* = 1,…, *n*_*i*_  *j* = 1,…, *k*, *x*_*ij*_ is a vector of individual-specific covariates, *β* is a vector of parameters, and *h*_*o*_ is the baseline hazard. Extending this model to include the spatial dependency, the likelihood for the Cox model was proposed with spatial and nonspatial frailty:

(s3) Lβ,W;t,x,γ∞∏j=1k∏i=1njhotijxijγij·exp⁡−Hotijexpβ′xij+wi+vi

where *w*_*i*_ is the spatial frailties and *v*_*i*_ is nonspatial frailties for the parameter. The event of interest was the death of MDR-TB patients during treatment as well as follow-up period. The event was coded as 1 for death occurring by TB and 0 for censor. There are other important covariates that are included in the analysis. The hospitals sites were the random effects for this study. The model distribution supports the possibility of correlated random effects with *W*_*i*_ ~ *N*(0, 1/*τ*), V represents non-frailty random effect, and this is exchangeable prior *V*_*i*_ ~ *N*(0, 1/*τ*).

#### 2.7.1. Weibull Model with Spatial Frailties

The joint posterior distribution for the spatial frailty parametric Weibull model is

(s4) pβ,W;ρ,x,λ ∣ t,x,γ∞Lβ,W,ρ;t,x,γpWλ·pβpρpλ

where *ρ* is the shape parameter for the baseline hazard in the Weibull model. The likelihood for the Bayesian-Weibull model with spatial individual frailties is as follows.

(s5) Lβ,W,ρ;t,x,γ∞∏i=1iρtiρ−1·exp⁡βTxi+wiγiexp⁡−tiρexp⁡βTxi+wi

The individual Weibull frailties are completed by assigning suitable prior for the parameter. Bayesian-Weibull model with spatial individual frailties is

(s6) Lβ,W,ρ;t,x,γ∞∏i=1iρtiρ−1·exp⁡βTxi+wiγiexp⁡−tiρexp⁡βTxi+wi+vi

where Vi represents the nonspatial frailties with *V*_*i*_ ~ *N*(0,1/*τ*)

## 3. Results

In this study, 207 multidrug resistance tuberculosis patients were considered to identify the factor of different duration death occurrence. Of these 61 (29.47%) were died and the rest, 146 (70.53%), of the patients were censored at the time of the study. Out of 207 MDR-TB patients, 146 (70.53%) were males, and 61 (29.5%) were females (Table 1).

The minimum duration of follow-up was one month whereas the maximum duration was 42 months.  Table 3 shows that the mean duration of death for MDR-TB patients was 31.907 month. The 95% confidence interval of mean duration of MDR-TB patients treated at hospital lies between 29.755 and 34.059 months. From 207 MDR-TB patients as therapeutic delay, 35.75% were started treatment after one month of diagnosis and the remaining 64.25% were started before one month of diagnosis. About 16.5% of MDR-TB patients were infected by HIV and 83.5% of them were HIV negative patients (Table 2).

To identify sets of covariates that have the potential influence to be included in the linear components of a multivariable model, univariate analysis was done. Covariates that were found to be significant in the univariable analysis were included in the multivariable analysis. We performed multivariable survival analysis by assuming exponential, Weibull, Gompertz, and Log logistic distributions for baseline hazard functions and the frailty distributions.

The AIC and BIC values of Weibull baseline distribution with frailty model are found to be minimum among all other considered models. The results indicated that Weibull baseline distribution with gamma frailty model is the most efficient model to describe the multidrug resistance tuberculosis (Table 4). Shared frailty model with Weibull baseline distribution has been given, which was found to be the best model for premature MDR dataset. The estimated values, standard error, accelerated factor, estimated parameters of baseline distributions, and frailty variance (*θ*) are presented in Table 5.

Age of MDR patient, therapeutic delay, alcohol user, any clinical complication, MDR category, HIV results, and chronic diseases were significant at 5 percent level of significance by using Weibull-gamma shared frailty model (Table 5).

Multidrug resistance tuberculosis patients with age difference was a significant factor for the death time of MDR. The hazard rate of death of MDR-TB patients who had age group of 55 and above year was 3.940 times higher than that of MDR-TB patients who had age group of 18-34 years (95% CI: 1.63, 9.549). Here, the confidence interval did not include one at 5% level of significance; they had the duration of death difference between ages group of MDR-TB. The age of MDR patients of 35-55 years was compared to 18-34 years and the accelerated factor was *HR* =1.823, 95%CI: 0.737, 4.503. Since the confidence interval includes one at 5% level of significance, the durations of death at the age group were statistically the same. Therefore, multidrug resistance tuberculosis patients from the 18-34-year age group had the longest duration of death compared to other age groups.

The therapeutic delay was a significant association with mortality of MDR-TB patients. The hazard ratio of death therapeutic delay before one month was 0.309 at 5% level of significance. The acceleration factor and 95% confidence interval for multidrug resistance tuberculosis were 0.309 and (0.166, 0.576), respectively. The estimated coefficient hazard ratio of death MDR-TB patient who starts treatment before one month was reduced by 61.0% compared to MDR-TB patient who starts treatment after one month.

The alcohol use was another prognostic factor that predicts the mortality of MDR-TB patients. The result of this study indicates that the hazard ratio of death of non-alcohol takers was 0.347 times that of alcohol user (HR = 0.347, 95% CI: 0.171, 0.702). This indicates that, in multidrug resistance tuberculosis patients, survivability of TB of alcohol users was shortened compared with non-alcohol users. The clinical complication was a determinant factor of multidrug resistance tuberculosis for time of death of patients. But Pneumonia, Pneumothorax complication, Hemoptysis, and other clinical complications were not statistically significant (Table 5). The hazard of death for MDR-TB patients with Cor pulmonary complication was 2.816 times higher than MDR-TB patients who did not develop any clinical complication (HR=2.816, 95% CI: 1.239, 6.403). These hazard ratios indicate that the risk of death of MDR-TB patients with Cor pulmonary complication is higher relative to MDR-TB patients who did not develop any clinical complication.

The estimated relative risk (hazard ratio) of death for MDR-TB patients who developed chronic disease varied. The hazard of death of MDR-TB patients who developed diabetes mellitus compared to those who did not develop chronic disease was 3.292 higher (95% CI: 1.329, 8.151). The duration of the death of MDR-TB patients who developed diabetes mellitus was higher than those with non-chronic disease. The hazard of death of MDR-TB patients with Myocardial infarction was 7.774 times higher than that of MDR-TB patients who did not develop chronic disease (95% CI: 2.904,20.812). This result revealed that the risk of death of MDR-TB patients with Asthma was 3.086 higher than that of MDR-TB patients with no chronic disease (95% CI 1.329, 17.162). Thus, the coinfected chronic disease was the risk factor for the death of MDR-TB patients. This indicated that the duration of death for MDR-TB patients who had coinfected chronic disease was shorter compared to MDR-TB patients free from any chronic disease.

The hazard of death of MDR-TB patients that were previously not treated as compared to those previously treated was higher. The hazard of death of those previously not treated was 2.329 (95% CI: 1.260, 4.307). This indicates that the hazard of death was higher for MDR-TB patients who are previously not treated relative to previously treated ones. The risk of death for MDR-TB patients infected by HIV could be higher than those non-infected by HIV (HR= 0.2021 (95% CI: 0.116, 0.387)). The value of the shape parameter in the Weibull-gamma frailty model was *ρ* = 1.687 which was greater than unity. This indicates that the shape of hazard function is unimodal; i.e., it increases for some time and then decreases. The variability (heterogeneity) in the population of clusters (districts MDR-TB by hospitals locations) estimated by our selected model was *θ* = 0.0000697, and the dependence within clusters was about *τ* = 0.0% (Table 5). The value of *θ* = 0.0000697 close to zero means no heterogeneity of death among groups (districted MDR-TB hospitals). The association between duration of mortality and patients' district was statistically insignificant (Table 5). This means the durations of patients' death at each district were the same.

## 4. Discussion

The main aim of the study was to determine survival time and predictors of mortality among patients under multidrug-resistant tuberculosis treatment. The study accounted for the correlation between MDR-TB among districts of hospitals. The comparison of models was selected by using the AIC and BIC criteria, where a model with minimum AIC and BIC was accepted to be the best. According to AIC and BIC, the Weibull-gamma shared frailty model was the most appropriate model to describe the multidrug resistance tuberculosis dataset.

Based on clustered district of multidrug resistance tuberculosis patients, no heterogeneity death occurred in patients. Hence, our study showed that there was no cluster (frailty) effect based on grouped district of the hospitals. The survival time of multidrug resistance tuberculosis variation was not due to the heterogeneity (among patients of the district). The district of hospitals did not effect the death of patients.

This study revealed that as the age of the patient's increases, the survival probability of the MDR-TB patient declines. Similar findings have been observed in [6, 7]. This research indicates that delays in diagnosis or treatment for MDR-TB patients result in short duration of mortality. The result obtained this study is that delays in diagnosis negatively impacted the duration of mortality in the MDR-TB patient. The finding of this study was similar to [7]. This study showed that alcohol user with multidrug resistance tuberculosis had a short duration compared to non-alcohol user. The result obtained from this study shows that alcohol user and their duration of morbidity were similar to those in Botswana study [8]. Multidrug resistance tuberculosis patients who were previously not treated had a relatively shorter duration than previously treated MDR-TB patients. This finding is consistent with the result reported by [7]. The hazard values of this study indicated that the risk of death of MDR-TB patients with different clinical complication was higher relative to MDR-TB patients with nonclinical complication. This is in agreement with some studies [6, 9].

Multidrug resistance tuberculosis patients having coinfected by HIV had shorter duration of death in treatment periods than that of HIV negative MDR-TB patients. This finding was consistent with a study in America and southern Africa [10, 11]. These findings were consistent with earlier studies [12, 13]. HIV and tuberculosis (TB) are so closely connected that their relationship is often described as a co-epidemic. In the last 15 years, the number of new TB cases has been more than doubled in countries where the number of HIV infections is also high [14]. The hazard ratios indicate that the risk of death of MDR-TB patients with different chronic coinfection is higher relative to MDR-TB patients with no chronic coinfection. This finding was consistent with [15] and the finding was similar to the previous finding from the state of Georgia and Southeastern Mexico [16, 17].

## 5. Conclusions

Based on AIC and BIC values, the most appropriate model for our dataset was Weibull, which well described the survival of tuberculosis. In this study, there was no frailty (district) effect on the survival of multidrug-resistant tuberculosis. The death of tuberculosis patients attending multidrug resistance was 61 (29.5%) and the rest, 146 (70.53%), of patients were censored. The mean duration of death attending tuberculosis in multidrug resistance was 31.07 months (95%, CI: 29.75, 34.056). The Weibull non-frailty model shows that the old age, delays treatment, alcohol use, any clinical complication previously not treated, positive HIV/AIDS, and any chronic disease of patients under multidrug resistance tuberculosis were significant variables.

## Figures and Tables

**Table 1:** Summary results of MDR–TB by different demographic characteristics.

Covariates	Category	Death (%)	Censored (%)	Total (%)
Sex	Female	33(40.74)	48(59.26)	81
	Male	28(22.22)	98(77.78)	126
Age	18-34 years	15(14.56)	88(85.44)	103
	35-54 years	22(39.3)	34(60.7)	56
	>= 55 years	24(50)	24(50)	48
Marital status of the patient	Single	16(20.78)	61(79.22)	77
	Married	36(36)	64(64)	100
	Separated/Divorced	6(31.58)	13(68.42)	19
	Widow/Widowed	3(30)	7(70)	10
Employment status	Employed	7(30.13)	16(69.56)	23
	Own Business	8(20)	32(80)	40
	Merchant	16(40)	24(60)	40
	Daily labor	4(23.53)	13(76.47)	17
	Unemployed	26(29.89)	61(70.11)	87
The educational level	Illiterate	16(25.81)	46(74.19)	62
	Read and Write	23(29.49)	55(70.51)	78
	Secondary	14(31.11)	31(68.88)	45
	Tertiary and above	8(36.36)	14(63.64)	22
Therapeutic delay*∗*	>= 1 Month	31(41.89)	43(58.11)	74
	< 1 Month	30(22.56)	103(77.44)	133
MDR category	Previously Treated for first-line TB	26(17.11)	126(82.89)	152
	Previously not Treated	35(64.81)	19(35.19)	54
Current Smoking Status	Yes	23(65.71)	12(34.29)	35
	No	38(22.09)	134(77.91)	172
Current Alcohol use	Yes	31(60.78)	20(39.22)	51
	No	30(19.23)	126(80.77)	156
City	Debre Tabor	6(27.27)	16(72.73)	22
	Gondar	49(33.56)	97(66.44)	146
	D/Markos	6(15.38)	33(84.62)	39

*∗* Therapeutic delay means patients first contact with health worker after 30 days (1 month) from the onset of TB symptoms.

**Table 2:** Summary results of MDR–TB by clinical characteristics.

Covariates	Category	Death (%)	Censored (%)	Total (%)
Any clinical complication	No complication	35(20.35)	137(79.65)	172
	Pneumonia	7(87.5)	1(12.5)	8
	Pneumothorax	6(85.71)	1(14.29)	7
	Hemoptysis	7(63.64)	4(36.36)	11
	Cor pulmonal	4(100)	0(0)	4
	Other	2(40)	3(60)	5
HIV Co-infection	Positive	21(61.76)	13(38.24)	34
	Negative	40(23.12)	133(76.88)	173
Acid-fast bacilli Smear (AFB)	positive	43(28.28)	109(71.71)	152
	Negative	12(30)	28(70)	40
Antibiotic Susceptibility	INH	6(72.86)	8(57.14)	14
	RMP	24(30.38)	55(69.62)	79
	MDR	26(30.95)	58(69.05)	84
	INH+RMP	5(16.67)	25(83.33)	30
Presence of any chronic disease	No chronic disease	42(24.14)	132(75.86)	174
	Diabetes Mellitus	8(50)	8(50)	16
	Myocardial infarction	4(100)	0(0)	4
	Asthma	4(66.66)	2(33.33)	6
	other	1(33.33)	2(66.67)	3
Radiological findings	unilateral cavity	9(26.47)	25(73.53)	34
	unilateral infiltration	3(33.33)	6(66.67)	9
	Bilateral cavity	4(18.18)	18(81.82)	22
	Bilateral inflation	7(38.89)	11(61.12)	18
	Non cavity	14(27.45)	37(72.55)	51
	Effusion	12(37.5)	20(62.5)	32
Smear positivity	Positive	46(31.94)	98(68.06)	144
	Negative	14(26.42)	39(73.58)	53
Clinical Presentation	Pulmonary	51(30)	119(70)	170
	Extra pulmonary	8(28.57)	20(71.43)	28

INH: isoniazid; RMP: rifampicin; MDR: multidrug-resistant.

**Table 3:** The mean duration of death for MDR TB patients.

Mean		95% confidence interval	
Estimate	Std. Error	Lower Bound	Upper Bound
31.907	1.098	29.755	34.059

**Table 4:** AIC and BIC values of shared frailty models.

Baseline Distributions	AIC	BIC
Exponential	251.7942	311.696
Gompertz	238.0642	301.2938
Log logistic	241.6048	304.8344
Weibull	234.4422	297.6718
Lognormal	249.4659	312.6955

**Table 5:** Model with shared frailty for multidrug resistance tuberculosis patients.

		Weibull (No frailty)				Weibull (Gamma)				Weibull (Inverse Gaussian)			
Variable	Category	HR	SE	P>z	95%CI *HR*	HR	SE	P>z	95%CI *HR*	HR	SE	P>z	95%CI *HR*
Age	18-34												
	35-54 years	1.823	0.841	0.193	[0.737, 4.503]	1.833	.841	0.193	[.738,4.502]	1.824	.843	0.194	[.737,4.514]
	>= 55 years	3.940	1.768	0.002	[1.635, 9.493]	3.939	1.77	0.002	[1.63, 9.491]	3.943	1.77	0.002	[1.63,9.516]
Therapeutic delay	< 1 Month	0.309	.0982	0.000	[0.166, 0.576]	.3092	.098	0.000	[.166, .5763]	.3095	.098	0.000	[.166,.577]
Alcohol use	No	0.347	.1247	0.003	[0.171, 0.702]	.3466	.125	0.003	[.171,.7017]	.3479	.125	0.003	[.172,.705]
Any clinical complication	No complication												
	Pneumonia	2.033	.9656	0.135	[0.802, 5.157]	2.033	.965	0.135	[.802,5.157]	2.037	.969	0.135	[.802,5.176]
	Pneumothorax	1.876	1.057	0.264	[0.622,5.659]	1.876	1.066	0.264	[.622,5.659]	1.874	1.06	0.266	[.620,5.666]
	Hemoptysis	1.524	1.008	0.524	[0.417,5.569]	1.524	1.007	0.524	[.417,5.568]	1.525	1.01	0.524	[.416,5.590]
	Cor pulmonale	2.816	1.180	0.013	[1.239,6.403]	2.816	1.180	0.013	[1.24,6.402]	2.822	1.18	0.013	[1.24,6.426]
	Other	4.11e-06	.0037	0.989	0	9.46e-11	.0001	1.000	0.	2.76e-08	.001	0.999	0
MDR category	Previously not Treated	2.329	.7306	0.007	[1.260,4.307]	2.329	.7305	0.007	[1.26,4.307]	2.329	.732	0.007	[1.26,4.311]
HIV	Negative	0.202	.0668	0.000	[0.110,0.387]	.2024	.0668	0.000	[.106,.3867]	.2023	.067	0.000	[.106,.3870]
chronic disease	No chronic disease												
	Diabetes Mellitus	3.292	1.523	0.010	[1.329,8.151]	3.292	1.523	0.010	[1.33,8.150]	3.294	1.53	0.010	[1.333,8.176]
	Myocardial infarction	7.774	3.906	0.000	[2.904,20.812]	7.773	3.905	0.000	[2.90,20.81]	7.774	3.913	0.000	[2.899,20.85]
	Asthma	3.085	1.326	0.009	[1.329,7.162]	3.085	1.325	0.009	[1.33,7.161]	3.086	1.328	0.009	[1.327,7.174]
	DM and HTN	2.483	2.980	0.449	[0.236,26.096]	2.483	2.980	0.449	[.236,26.09]	2.498	2.999	0.446	[.237,26.280]
	Other	1.950	2.162	0.547	[0.223,17.139]	1.949	2.162	0.547	[.223,17.13]	1.956	2.171	0.545	[.222,17.225]

	_cons	0.0045	.0032	0.000	[0.001,0.018]	.0045	.0032	0.000	[.001,.0184]	.0044	.0032	0.000	[.001,.018]
	/ln_p	0.5228	.1066	0.000	[0.314,0.73]	.5228	.1066	0.000	[.314,.7318]	.5231	.1072	0.000	[.313,.733]
	/ln _the					-16.82	849.2	0.984	[-168, 1647]	-9.87	26.49	0.709	[-61.788,42.04]
	P	1.687	0.1799		[1.369,2.079]	1.687	0.1798		[1.367,2.08]	1.687	.1809		[1.367,2.082
	1/p	0.5929	.0633		[0.482,0.731]	0.5928	.0632		[0.480,0.731]	.5927	.0636		[0.480,0.731]
	Theta					0.0000697	.002		[2.11e-24 2.30e+15]	0.0000516	0.0014		[1.46e-27 1.82e+18]

## Data Availability

In consultation with the Bahir Dar University Ethics Committee that approved this study, the data of the study cannot be publicly available due to the privacy protection of patients. Therefore, sharing the dataset is not possible.

## Abbreviations

AFB: Acid-fast bacilli smear
AIC: Akaike information criterion
BIC: Bayesian information criterion
CI: Confidence interval
HR: Hazard ratio
MDR: Multidrug resistance
TB: Tuberculosis
HIV: Human immunodeficiency virus
WHO: World Health Organization.

## Additional Points

Multiple drug resistance (MDR) is antimicrobial resistance shown by a species of a microorganism to multiple antimicrobial drugs [18]. Acid-fast bacilli (AFB) smear and culture are two separate tests always performed together at the MSPHL, Tuberculosis (TB) Unit. AFB smear refers to the microscopic examination of a fluorochrome stain of a clinical specimen. The AFB culture is the inoculation of a clinical specimen onto culture media Becton-Dickinson Mycobacteria Growth Indicator Tube (B-D MGIT broth) and Lowenstein-Jensen (L-J) media slant, incubation at 37°C for up to six (6) weeks, and detection of growth or no growth during this incubation period [19]. Antibiotic sensitivity or antibiotic susceptibility is the susceptibility of bacteria to antibiotics. Smear-positive TB case, according to WHO definition of a new sputum smear-positive pulmonary TB case, is based on the presence of at least one acid-fast bacilli (AFB+) in at least one sputum sample in countries with a well-functioning external quality assurance (EQA) system.
